# EndoBarrier®: a Safe and Effective Novel Treatment for Obesity and Type 2 Diabetes?

**DOI:** 10.1007/s11695-018-3123-1

**Published:** 2018-02-15

**Authors:** Nisha Patel, Aruchuna Mohanaruban, Hutan Ashrafian, Carel Le Roux, James Byrne, John Mason, James Hopkins, Jamie Kelly, Julian Teare

**Affiliations:** 1 St Mary’s Hospital, Imperial College London, Praed Street, London, W2 1NY UK; 2 Metabolic Medicine, Charing Cross Hospital, Imperial College London, Fulham Palace Road, London, W6 8RF UK; 3 University Hospital Southampton NHS Foundation Trust, Southampton General Hospital, Tremona Road, Southampton, Hampshire, SO16 6YD UK; 4 Trafford Hospitals, Central Manchester University Hospitals NHS Foundation Trust, Moorside Road, Davyhulme, Manchester, M41 5SL UK; 5 North Bristol NHS Trust, Southmead Hospital Bristol, Southmead Road, Westbury-on-Trym, Bristol, BS10 5NB UK

**Keywords:** Obesity, Type 2 diabetes, Bariatric surgery, Bypass surgery, Duodenal-jejunal sleeve

## Abstract

**Background and Aims:**

Obesity associated with diabetes mellitus is a significant worldwide problem associated with considerable health care costs. Whilst surgical intervention is effective, it is invasive, costly and associated with complications. This study aims to evaluate the safety and efficacy of the EndoBarrier®, a duodenal-jejunal sleeve bypass as an alternative treatment of diabetes mellitus in obese patients.

**Materials and Methods:**

This was a multi-centre, non-randomised trial recruiting obese patients with type 2 diabetes from three sites in the UK. Eligible participants had a BMI of 30–50 kg/m^2^ and HbA1c levels of 7.5–10%. The study comprised a 12-month period with the EndoBarrier® inserted and a 6-month follow-up period after it had been explanted. The primary study outcomes were weight, BMI, HbA1c levels and fasting insulin and glucose levels.

**Results:**

Forty-five patients were recruited and 31 patients (69%) completed the 12-month study period. Significant reductions in weight (95%CI 0.62–29.38; *p* < 0.05) and BMI (95%CI 1.1–8.7; *p* < 0.005) were documented 12 months after device insertion*.* The mean HbA1c was significantly reduced (95%CI 0.1–1.6; *p* < 0.05) after the device insertion period and reductions in metabolic parameters (fasting insulin and glucose levels) were also documented during the study. Adverse events were also assessed in all patients, the vast majority of which were reported as mild.

**Conclusions:**

The EndoBarrier® appears to be a safe and effective treatment strategy in overweight patients with poor glycaemic control despite medical therapy, or in those who are eligible but decline bariatric surgery.

## Introduction

Obesity is a modern pandemic. One of the major complications of obesity is the development of diabetes; the US Centre for Disease Control found that 85% of newly diagnosed type 2 diabetics were overweight or obese and 54% obese [1]. Obesity-associated diabetes mellitus leads to considerable mortality, morbidity and enormous health care costs. In the UK, obesity is estimated to consume 1.5% of the NHS budget with an additional 10% of the budget spent on diabetes care. In the USA, medical costs directly related to diabetes complications for the year 2006 were estimated at $22.9 billion. It is thus a critical concern for global health care.

Medical therapy to control diabetes can be disappointing. Only half of patients with diabetes achieve a satisfactory control measured by the glycosylated haemoglobin levels (HbA1c) on medical treatment, whilst surgical interventions are more effective, but are not widely provided [2]. At present, surgery is the only treatment that delivers sustainable weight loss and glycaemic control [3, 4] in obese patients. Hyperglycaemia can improve within days of surgery and before significant weight loss has occurred [5], suggesting the role of weight-independent mechanisms of enhancing glucose metabolism.

The Roux-en-Y gastric bypass (RYGB) operation was first performed as a bariatric procedure in the 1960s[6] (minimally invasively/laparoscopically since the 1990s) and delivers sustained weight loss and resolution of obesity-related comorbidities, including type 2 diabetes [7]. In view of its clinical efficacy, it remains among the most preferred bariatric procedures in obese patients with type 2 diabetes mellitus. Following RYGB, ingested food enters the jejunum via a small stomach pouch, thereby bypassing the majority of the stomach, the duodenum and the proximal 25–50 cm of the jejunum, and achieves a number of immediate physiological effects. These include bile flow alteration, reduction of gastric size, anatomical gut rearrangement and alteration of flow of nutrients, vagus nerve manipulation and enteric gut hormone shifts (the so-called BRAVE effects) that subsequently result in several downstream effects such as microbiota modulation, adipokine release and alteration in glucose metabolism. Gastric bypass can be performed with a low 30-day in-hospital mortality rate of 0.3% [8]. The superior effects of RYGB in initial weight loss compared with gastric banding and sleeve gastrectomy are postulated to be due to its multiple physiological effects. These include reduced hunger, increased satiety and, as more recently described from pre-clinical and clinical studies, beneficial effects on taste and food preference away from high-calorie foods [9, 10]. Whilst bariatric surgery can be performed safely, there is a higher complication risk in patients with diabetes and or obesity by virtue of these conditions themselves, co-morbidities and their associated therapies such as antiplatelet medication. These include general post-surgical medical complications such as pneumonia and venous thrombosis (pulmonary embolism and deep vein thrombosis). Post-operative complications such as anastomotic disruption leading to leaks and fistulae [11], stomal stenosis [12], dumping syndrome and diarrhoea are also problematic.

The duodenal-jejunal sleeve bypass (DJSB) or EndoBarrier® (GI Dynamics Inc., Lexington MA) is an endoscopically implantable and removable device that is anchored in the first part of the duodenum, where it is attached by a nitinol stent anchor to a 60-cm long polymer sleeve. The sleeve prevents ingested food from coming into contact with the mucosa of the proximal upper intestine. The design of the DJSB therefore offers functional similarities to mimic some of the physiological effects of Roux-en-Y gastric bypass. These include exclusion of food from the proximal small intestine and mixing of pancreatic and biliary juices after food passes through the sleeve. Initial studies in humans have been for a 6-month implant duration, with 6 months’ follow-up data, and have also shown between 11.9 and 23.6% of total bodyweight loss [13, 14] with improvements in diabetes including reduction in HbA1c and normoglycaemia [15, 16].

The aim of this prospective study was to assess the safety and efficacy of the EndoBarrier® in obese patients with type 2 diabetes over an 18-month period, with a 12-month implant duration, and then 6 months follow-up.

## Materials and Methods

### Study Design and Participants

This was a non-randomised study conducted at three investigational sites (Southampton, London and Manchester) in the UK to determine the performance of the EndoBarrier® for the treatment of obesity and type 2 diabetes. The study was conducted in accordance with Standard ISO 14155:2003 on clinical investigations with medical devices as well as the Helsinki Declaration. Ethics committee approval was granted prior to commencement of the study and any protocol deviations reported. All patients provided written informed consent.

### Recruitment

Subjects were recruited from hospital and community-based diabetes clinics. Eligible study participants aged between 18 and 65 years with a history of diabetes and duration of 1–10 years were invited to an initial assessment. This comprised of baseline measures including demographic data, diabetes and medical history and medication history. Baseline blood tests including HbA1c, insulin and lipid profile were also performed.

### Key Inclusion and Exclusion Criteria

Participants who fulfilled the inclusion criteria and none of the exclusion criteria were enrolled into the study (Table 1). The key inclusion criteria included:

- Subjects with HbA1c level of 7.5 to 10% (58.5–85.5 mmol/mol)
- Subjects with a BMI of 30 to 50 kg/m^2^
- Subjects taking oral type 2 diabetic medication and/or insulin

**Table 1:** Baseline characteristics for the implanted population

Subjects (*n* = 45)	
Age, mean ± SD, years	49.9 ± 7.9
Gender, *n* (%)	
Male	22 (48.9)
Female	23 (51.1)
Race: Caucasian, *n* (%)	40 (88.9)
Weight, mean ± SD, kg	115.0 ± 21.0
BMI, mean ± SD, kg/m^2^	40.0 ± 5.8
HbA1c, mean ± SD (%)	8.5 ± 0.8
Duration of diabetes, mean ± SD, years	4.6 ± 2.8
Glucose, mean ± SD, mmol/L	9.5 ± 2.95
Insulin, mean ± SD, mIU/L	18.8 ± 10.41
Total cholesterol, mmol/L	4.3 ± 0.97
Systolic BP, mmHg	141 ± 20
Diastolic BP, mmHg	82 ± 10
Comorbidities, *n* (%)	
Hypertension	29 (64.4)
Hyperlipidaemia	32 (71.1)
Coronary artery disease	1 (2.2)
Sleep apnoea	3 (6.7)

The key exclusion criteria included:

- Subjects requiring > 150 units of insulin/day
- Subjects with fasting C peptide serum < 1.0 ng/mL
- Subjects taking DPP4 inhibitors or incretins e.g. sitagliptin or exenatide
- Subjects with type 1 diabetes or history of ketoacidosis

Once written consent was obtained, subjects were enrolled in a nutritional counselling programme delivered by specialist dieticians. The aim of the nutritional counselling programme was to provide study participants with lifestyle and behaviour modification, an understanding of calorific intake limitation and good eating practices. Subjects were given advice regarding liquid/modified diet for the first few weeks following implantation. Subjects were tested for *Helicobacter pylori* and if positive were prescribed eradication therapy. Subjects were prescribed a proton pump inhibitor (omeprazole 40 mg BID) to be taken from 3 days prior to device insertion and then continued until 2 weeks after explant.

### Study Period

The study period was 18 months in total; study participation was considered complete at this point. Clinical and biochemical assessments were carried out at baseline then 3, 6, 9 and 12 months and then post-device insertion. Device insertion was performed under general anaesthetic with X-ray screening. Explant was performed either under general anaesthetic or sedation and with X-ray screening at 12 months. Further assessments were then conducted at 15 and 18 months—for 3 and 6 months post-explant (PE) with body mass index (BMI), medication history and blood tests taken at each visit. Patients were also asked to fill out a health questionnaire at the start and end of the study. Details regarding health in general and limitations of activity due to physical and emotional health were documented. An assessment of mood, pain and sequelae of physical or emotional problems was reported. All authors had access to the study data and have reviewed and approved the final manuscript.

### Diabetes Management

Study subjects had their dose of insulin and sulphonylureas reduced by 50% at the time of insertion to avoid hypoglycaemic episodes. Metformin doses were reduced by 50% only if the fasting glucose was less than 3.9 mmol/L. Re-introduction or dose modifications of diabetic medication were made at the local study investigator’s discretion if inadequate glycaemic control during the study was noted.

The study investigators assessed the subjects for adverse events throughout the study including unscheduled visits. Subjects were withdrawn from the study if they, the sponsor or the investigator requested it, if the subject was lost to follow-up or if an adverse event required device explant and study withdrawal.

### Adverse Events

Safety measures were reported by evaluating the incidence and severity of adverse events at each study assessment visit. The adverse event, system it affected e.g. gastrointestinal tract, dates of onset and resolution were documented. The severity of the event was established and agreed by both patient and clinician. Details regarding unscheduled gastroscopies, changes in medication, other medical treatments and all hospital admissions were also determined. Severe adverse events were recognised to be a serious deterioration in the subject’s health and included hospital admissions, medical or surgical intervention and life-threatening conditions.

### Statistical Analysis

Statistic and data syntheses were performed in Microsoft Excel for Mac Version 14.4.4 (Microsoft Corporation, Redmond, WA, USA) and Stata Version 12 (Stata Corp LP, TX, USA).

## Results

### Study Population

A total of 45 subjects were enrolled into the study; 31 patients (69%) completed the 12-month study period. A summary of baseline characteristics and subject demographics is shown in Table 1. A list of patient co-morbidities was obtained from their general practitioner including date of diabetes onset and prescribed medication.

### Device Insertion Period

**Fig. 1:**
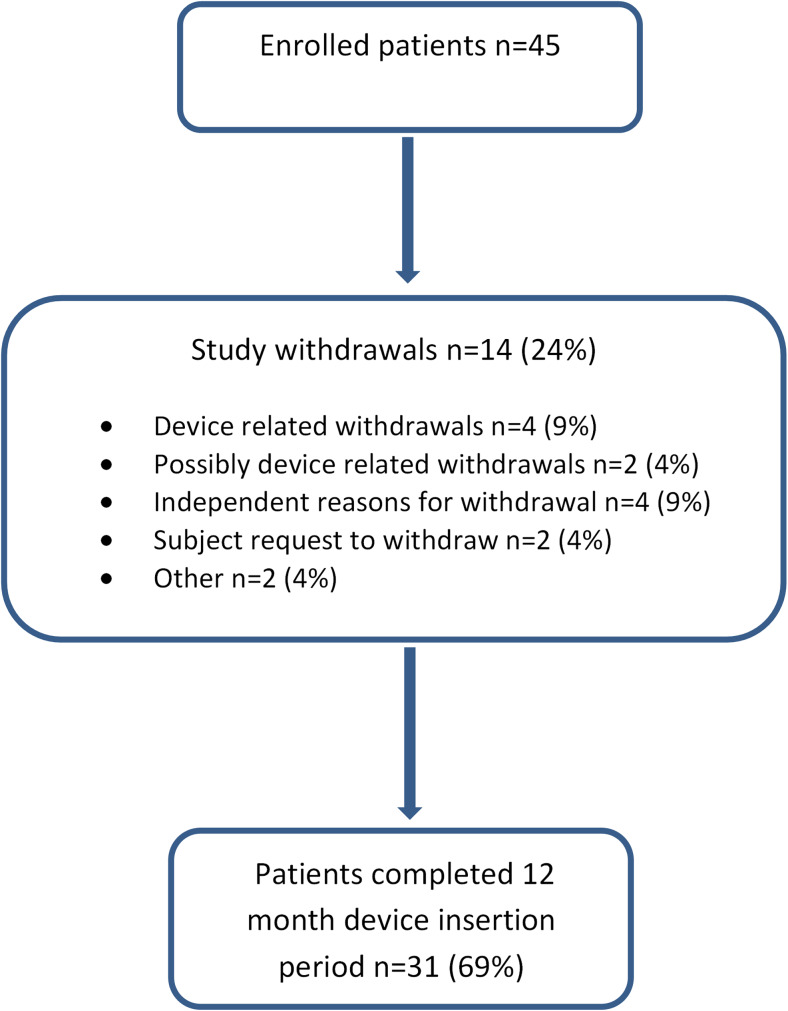
Flow chart demonstrating the number of patients involved in the study and study withdrawals

The device was successfully inserted in all cases; the mean insertion time was 27 min and fluoroscopic time 7 min. There were no procedure-related complications at insertion and all devices were explanted successfully (Fig. 1). There was one explant-related complication described below relating to the endoscope cap which was resolved without removal of the device and with no subsequent complications.

### Adverse Events

Forty of the 45 study patients (88.9%) reported 127 device-related adverse events from their individual completed study period (Table 2). The vast majority of patients (38 (84.4%)) experienced mild device-related events. Table 2 outlines all adverse events (described as mild, moderate or severe) experienced by study participants, with detailed reporting on gastrointestinal disturbances such as abdominal pain or discomfort, constipation, diarrhoea and dyspepsia which accounted for most of the device-related adverse events. Thirty-eight patients (84.4%) experienced at least one gastrointestinal adverse event: ten patients (22.2%) reported 12 procedure-related adverse events, 7 (15.6%) of which were classified as mild including nausea, vomiting and pharyngolaryngeal pain.

**Table 2:** Adverse events classed by system or organ with an expansion on gastrointestinal disorders reported from all 45 study participants

Adverse events by system or organ	Device	Procedure	Non-device or procedure	Pre-existing	At least 1 adverse event experienced
	*n* (%)	*n* (%)	*n* (%)	*n* (%)	*n* (%)
Blood and lymphatic system disorders e.g. anaemia	5 (11.1)	0 (0.0)	0 (0.0)	0 (0.0)	5 (11.1)
Cardiac disorders e.g. myocardial infarction	0 (0.0)	0 (0.0)	2 (4.4)	0 (0.0)	2 (4.4)
Gastrointestinal disorders	32 (71.1)	3 (6.7)	14 (31.1)	2 (4.4)	38 (84.4)
Abdominal discomfort	7 (15.6)	0 (0.0)	0 (0.0)	0 (0.0)	7 (15.6)
Abdominal distension	1 (2.2)	0 (0.0)	0 (0.0)	0 (0.0)	1 (2.2)
Abdominal pain	5 (11.1)	0 (0.0)	2 (4.4)	0 (0.0)	7 (15.6)
Abdominal pain lower	1 (2.2)	0 (0.0)	2 (4.4)	0 (0.0)	3 (6.7)
Abdominal pain upper	13 (28.9)	3 (6.7)	0 (0.0)	2 (4.4)	18 (40.0)
Colonic polyp	0 (0.0)	0 (0.0)	1 (2.2)	0 (0.0)	1 (2.2)
Constipation	4 (8.9)	0 (0.0)	2 (4.4)	0 (0.0)	6 (13.3)
Diarrhoea	4 (8.9)	0 (0.0)	5 (11.1)	0 (0.0)	9 (20.0)
Duodenal ulcer	1 (2.2)	0 (0.0)	0 (0.0)	0 (0.0)	1 (2.2)
Duodenitis	0 (0.0)	0 (0.0)	1 (2.2)	0 (0.0)	1 (2.2)
Dyspepsia	4 (8.9)	0 (0.0)	1 (2.2)	0 (0.0)	5 (11.1)
Epigastric discomfort	1 (2.2)	0 (0.0)	0 (0.0)	0 (0.0)	1 (2.2)
Flatulence	3 (6.7)	0 (0.0)	0 (0.0)	0 (0.0)	3 (6.7)
Food poisoning	0 (0.0)	0 (0.0)	2 (4.4)	0 (0.0)	2 (4.4)
Gastroesophageal reflux disease	0 (0.0)	0 (0.0)	2 (4.4)	0 (0.0)	2 (4.4)
Irritable bowel syndrome	1 (2.2)	0 (0.0)	0 (0.0)	0 (0.0)	1 (2.2)
Melaena	1 (2.2)	0 (0.0)	0 (0.0)	1 (2.2)	2 (4.4)
Nausea	5 (11.1)	0 (0.0)	3 (6.7)	0 (0.0)	8 (17.8)
Oesophageal polyp	0 (0.0)	0 (0.0)	1 (2.2)	0 (0.0)	1 (2.2)
Rectal haemorrhage	1 (2.2)	0 (0.0)	0 (0.0)	0 (0.0)	1 (2.2)
Reflux gastritis	0 (0.0)	0 (0.0)	2 (4.4)	0 (0.0)	2 (4.4)
Stomach discomfort	1 (2.2)	0 (0.0)	0 (0.0)	0 (0.0)	1 (2.2)
Vomiting	7 (15.6)	0 (0.0)	1 (2.2)	0 (0.0)	8 (17.8)
General disorders e.g. pyrexia, fatigue	7 (15.6)	1 (2.2)	4 (8.9)	1 (2.2)	12 (26.7)
Hepatobiliary disorders	1 (2.2)	0 (0.0)	2 (4.4)	0 (0.0)	3 (6.7)
Infections and infestations e.g. cellulitis	1 (2.2)	0 (0.0)	12 (26.7)	0 (0.0)	13 (28.9)
Injury, poisoning and procedural complications e.g. vomiting	10 (22.2)	1 (2.2)	5 (11.1)	0 (0.0)	15 (33.3)
Metabolism and nutrition disorders e.g. iron deficiency	18 (40.0)	0 (0.0)	1 (2.2)	0 (0.0)	19 (42.2)
Musculoskeletal and connective tissue disorders	7 (15.6)	1 (2.2)	15 (33.3)	1 (2.2)	21 (46.7)
Blepharal papilloma	0 (0.0)	0 (0.0)	1 (2.2)	0 (0.0)	1 (2.2)
Nervous system disorders e.g. CVA	1 (2.2)	0 (0.0)	3 (6.7)	0 (0.0)	4 (8.9)
Psychiatric disorders e.g. depression	0 (0.0)	0 (0.0)	5 (11.1)	1 (2.2)	6 (13.3)
Renal and urinary disorders	0 (0.0)	0 (0.0)	1 (2.2)	0 (0.0)	1 (2.2)
Reproductive system and breast disorders	1 (2.2)	0 (0.0)	1 (2.2)	0 (0.0)	2 (4.4)
Respiratory, thoracic and mediastinal disorders e.g. apnoea	0 (0.0)	3 (6.7)	3 (6.7)	0 (0.0)	6 (13.3)
Skin and subcutaneous tissue disorders	2 (4.4)	0 (0.0)	1 (2.2)	0 (0.0)	2 (4.4)
Vascular disorders e.g. DVT	1 (2.2)	2 (4.4)	1 (2.2)	2 (4.4)	6 (13.3)

Fourteen patients reported serious or severe adverse events during the study period, all of which had completely resolved at the end of the study. Of these, four patients complained of severe abdominal pain and were admitted between 2 and 4 days. One patient was treated for constipation with laxatives, one patient was found to have gallstones and required a cholecystectomy and one was admitted for observation after a normal gastroscopy. The final patient with abdominal pain was found to have a device that had migrated greater than 3 cm and hence had the device explanted. One patient was found to have upper gastrointestinal bleeding with erosions found on gastroscopy and required removal of the device. In one patient, the explant endoscope cap was lodged in the pharynx and required removal of the cap (but not the device) under general anaesthetic. This accounts for the only procedure-related event reported during the study.

There were eight patients with serious adverse events not relating to the study that required hospital admission. These were for conditions including atrial flutter, myocardial infarction, musculoskeletal pain, urinary tract infection, gout, deep vein thrombosis (DVT), respiratory compromise and possible stroke. In all these cases, the patient had complete resolution of the events.

### Study Withdrawals

A full data set was collated for all 45 patients at baseline. Thirty-one patients completed the 12-month study period; this data was included for analysis. Fourteen subjects withdrew from the study before 12 months; most of these did not attend subsequent appointments and were ultimately lost to follow-up (Table 3). The device was explanted early in 6 out of the 14 cases (Table 4). Two of these participants had device-related adverse events requiring premature explant for melaena and device migration resulting in abdominal pain respectively. The other participants developed the unavoidable complications of gout requiring non-steroidal anti-inflammatory medication and vascular complications including myocardial infarction, DVT and a transient ischaemic attack requiring anticoagulation or antiplatelet medication. Both of these are contraindications to the EndoBarrier® and hence resulted in device explant. The number of patients who withdrew from the study at different time intervals is shown in the table below.

**Table 3:** The number of participants and withdrawals at the main study intervals

Number of study months completed	Number of study participants involved in the study (%)	Cumulative number of study participants who withdrew from study (%)
0, study baseline	45 (100)	0 (0)
3	42 (93.3)	3 (6.7)
6	38 (84.4)	7 (15.5)
9	35 (77.8)	10 (22.2)
12, study completed	31 (68.9)	14 (31.1)

**Table 4:** Study withdrawals including early device explants

Study withdrawals/non-completers	Reason for withdrawal	Day of onset post device insertion	Severity	Device related or pre-existing/independent cause	Device explanted early
1	Intermittent upper abdominal pain	205	Severe	Device related	No
2	Abdominal pain	140	Moderate	Device related (device migration)	Yes
3	Atrial flutter	68	Moderate	Pre-existing/independent	No
4	Abdominal pain	28	Moderate	Possibly device related	No
5	Aggression	266	Mild	Other—due to omeprazole side effects	No
6	Possible transient ischaemic attack requiring aspirin	284	Moderate	Pre-existing/independent	Yes
7	Non-compliant with gliclazide	N/A	N/A	N/A	No
8	Gout	273	Severe	Pre-existing/independent	Yes
9	Myocardial infarction	22	Moderate	Pre-existing/independent	Yes
10	Subject requested to withdraw from study	N/A	N/A	N/A	No
11	Subject requested to withdraw from study	N/A	N/A	N/A	No
12	Abdominal pain	2	Moderate	Device related	No
13	Gastrointestinal bleeding	28	Moderate	Device related	Yes
14	Deep vein thrombosis	11	Moderate	Possibly related to device implant/explant procedure	Yes

Three subjects withdrew from the study before 3 months, three between 3 and 6 months, one between 6 and 9 months and three between 9 and 12 months due to adverse events. One patient withdrew due to side effects from omeprazole, one withdrew as they were non-compliant with gliclazide and two subjects requested to leave the study (Table 4).

The participant who did not complete the study due to non-compliance with gliclazide suffered from fatigue, continuous back pain and mild lower respiratory tract infection and cholelithiasis. All of these conditions were mild with no clear onset and only the fatigue was felt to be possibly device related. The two subjects who requested to withdraw from the study reported a number of mild to moderate adverse events including nausea, vomiting, constipation, abdominal pains and continuous rib and back pain that were felt to possibly be device related. One participant suffered a moderate rectal haemorrhage 197 days after the device was inserted and was felt to be device related; the other participant suffered a severe urinary tract infection that was felt to be possibly device related.

Thirteen out of the 14 patients who withdrew did not attend their 3-month PE or further appointments for data collection. Three more patients did not attend their 6-month PE appointment for data collection. Data was hence collected for a differing number of study subjects for both 3 and 6 months PE for each study indicator. Data from between 28–31 patients and 24–27 patients 3 and 6 months PE was available from the full data set and between 24–26 and 19–23 patients from the completer population 3 and 6 months PE respectively.

### Study Parameters

#### Weight and BMI

**Fig. 2:**
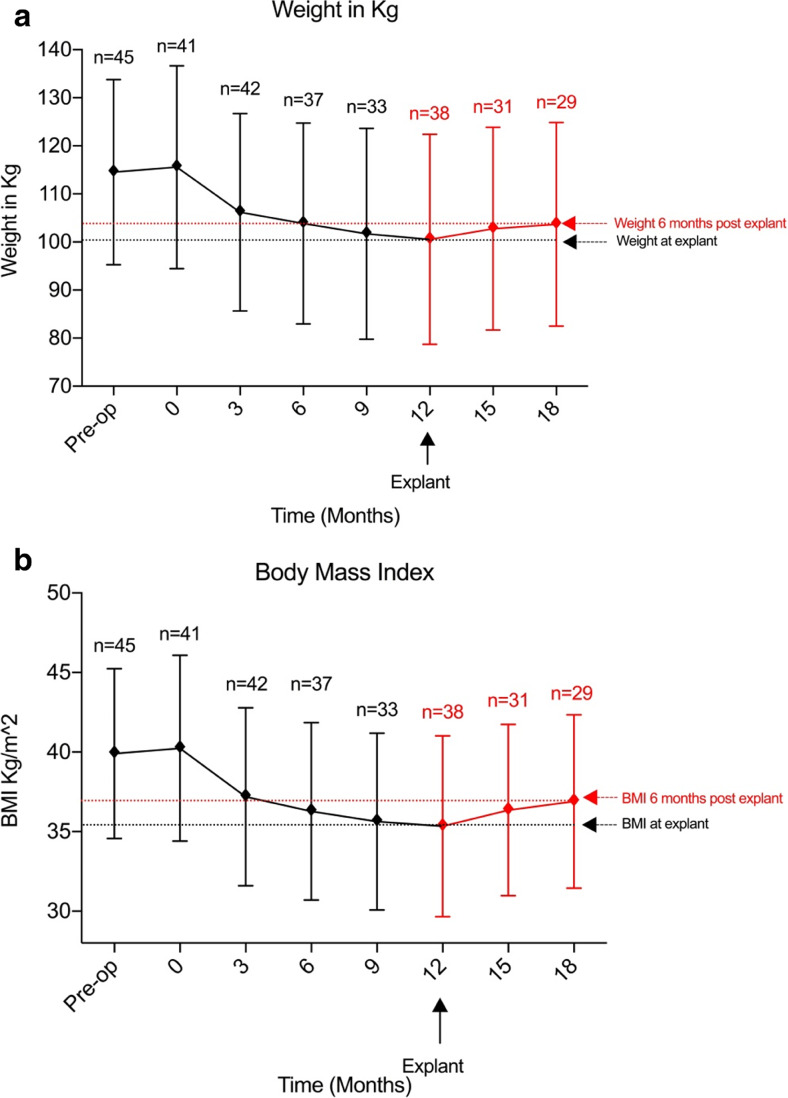
Weight (**a**) and BMI (**b**) measurements at all time points from baseline (pre-operative) to post-explant follow-up

During the device insertion period, both weight and BMI were significantly reduced from baseline values with both parameters at their lowest point 12 months after insertion. The mean weight loss was 15 kg (95%CI 0.62–29.38; *p* < 0.05) 12 months after the device was inserted. Weight loss was most rapid within the first 3 months of insertion (baseline 115.6 ± 21.1 kg (mean ± SEM), 3 months 106.2 ± 20.5 kg; difference 9.4 kg (95%CI − 4.6 to 23.4; *p* = ns)) (Fig. 2a).

BMI was reduced by 4.9 kg/m^2^ (95%CI 1.1–8.7; *p* < 0.005) 12 months after device insertion and by 4.6 kg/m^2^ (95%CI 0.8–8.3; *p* < 0.005) from baseline. Significant reductions in BMI from baseline to 6, 9 and 12 months were observed (mean difference 3.97 kg/m^2^ (95%CI 0.12–7.82; *p* < 0.05), 4.6 kg/m^2^ (95%CI 0.634–8.57; *p* < 0.05) and 4.90 kg/m^2^ (95%CI 1.08–8.71; *p* < 0.005) respectively) (Fig. 2b).

Following explant, weight increased by 2.2 ± 5.1 kg at 3 months and 3.1 ± 5.2 kg at 6 months (Fig. 2a). Non-significant increases from baseline BMI were noted at 3 and 6 months PE (increase of 1.0 kg/m^2^ (95%CI − 5.1 to − 3.1; *p* = ns) and 1.6 kg/m^2^ (95%CI − 5.7 to 2.6; *p* = ns) respectively). In addition, neither of these values were significant when compared with BMI at the time of explant (Fig. 2b).

#### HbA1c

**Fig. 3:**
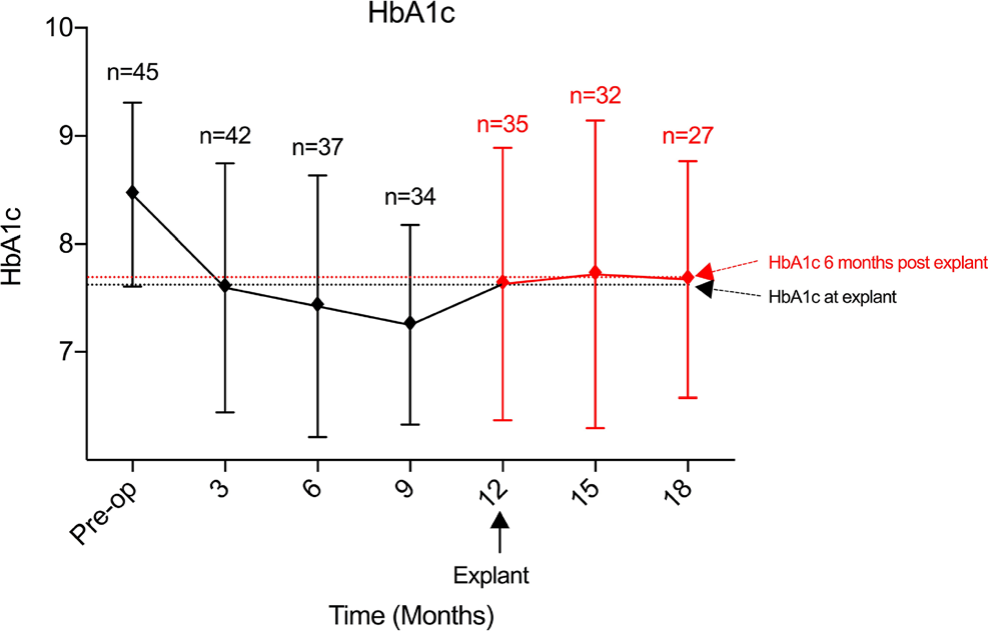
Measured values of HbA1c at all time points

HbA1c was significantly reduced from baseline values during the 12-month device insertion period. At 12 months, the mean HbA1c was 0.8% below the mean at baseline (95%CI 0.1–1.6; *p* < 0.05) (Fig. 3). A significant reduction in HbA1c was seen as early as 3 months after insertion (0.9% decrease (95%CI 0.1–1.6; *p* < 0.05)). This was further increased to 1.0% reduction at 6 months (*p* < 0.005) and 1.2% reduction at 9 months (*p* < 0.00001).

Following explant, HbA1c levels remained stable; the mean HbA1c was 7.7 ± 1.3% at 3 months and 7.7 ± 1.1% at 6 months. This equated to a mean difference from baseline of 0.7 ± 0.3% (*p* = ns) and 0.8 ± 0.3% (*p* = ns) (Fig. 3).

#### Fasting Plasma Insulin and Glucose

**Fig. 4:**
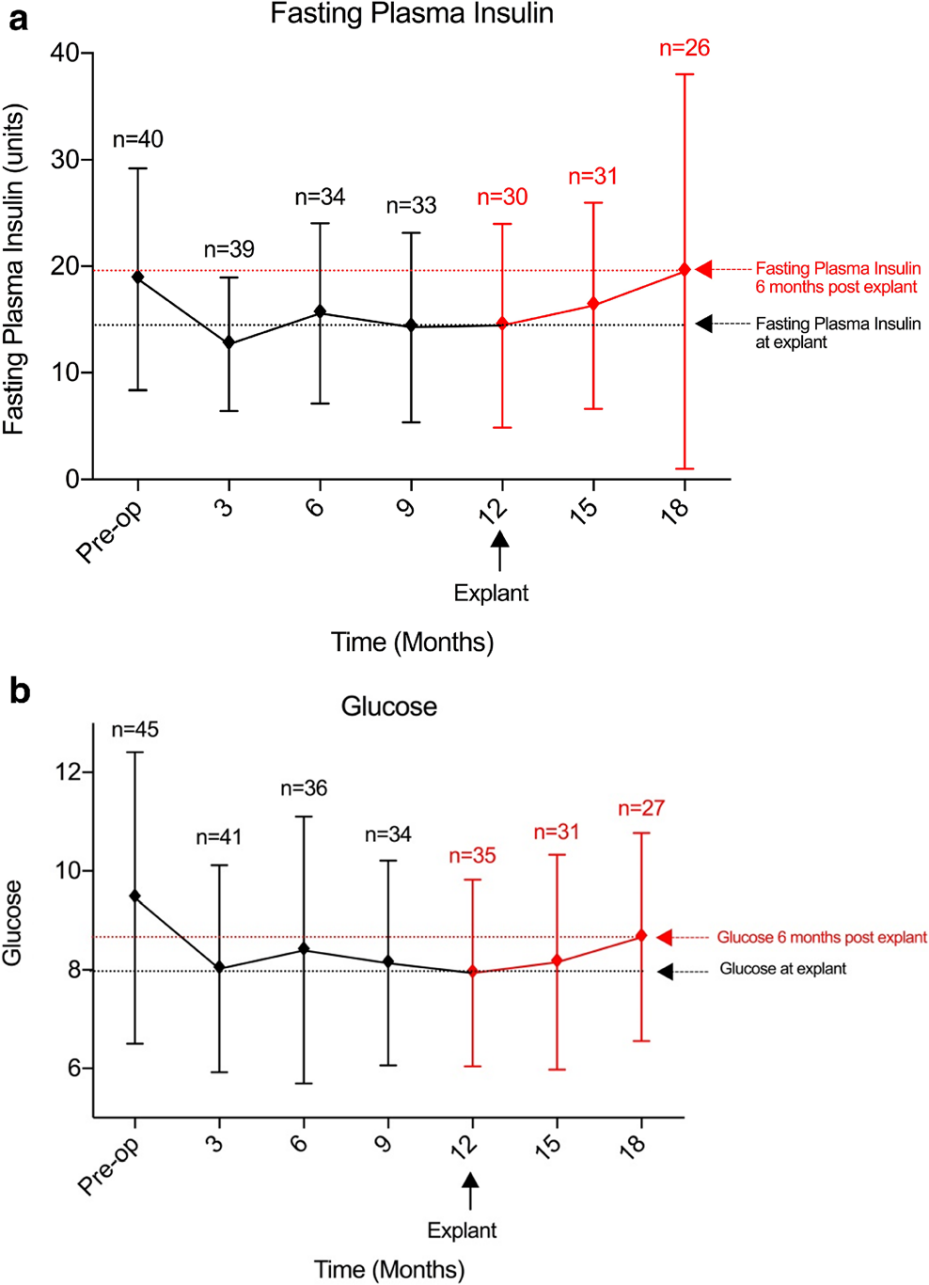
Fasting plasma insulin (miU/L) (**a**) and fasting plasma glucose (IU) (**b**) at all time points from baseline to post-explant follow-up

Reductions in both plasma insulin and glucose levels were observed during the device insertion period. Fasting plasma insulin levels had reduced by 4.4 mu/L (95%CI − 3.1 to 11.9; *p* = ns) (Fig. 4a) and fasting plasma glucose reduced by 1.5 mmol/L (95%CI − 0.1 to 3.1; *p* = ns) measured 12 months after insertion (Fig. 4b).

There was no change in fasting plasma insulin observed between baseline and 6 months post-explant 0.71 miU/L (95%CI − 8.53 to − 7.11; *p* = ns) (Fig. 4a). A reduction of 0.8 mmol/L (95%CI − 0.91 to 2.50; *p* = ns) in fasting glucose was observed from baseline to 6 months after explant (Fig. 4b).

### Changes in Medication

The majority of patients were taking metformin and sulphonylureas for their diabetes; one patient required insulin (Table 5).

**Table 5:** Baseline medication for diabetes

Diabetes medication at baseline	Number of patients (%)
Acarbose	1 (2.2)
Gliclazide	16 (35.6)
Glimepiride	2 (4.4)
Insulin	1 (2.2)
Metformin	40 (88.9)
Pioglitazone	4 (8.9)

Approximately 28% of patients taking metformin had their dose of the drug reduced or discontinued 6 months after device insertion (Table 6) which was similar after 12 months. Approximately 28% of patients taking sulphonylureas had their dose reduced or discontinued the drug 6 months after device insertion. This increased to 31% after 12 months.

**Table 6:** Changes in diabetic medication during the 12-month device insertion period

Change in diabetes medication	Months post-EndoBarrier® insertion	
	6 months	12 months
Sulphonylureas, *n* (%)	*n* = 36	*n* = 45
Increased	1 (2.8)	1 (2.2)
No change	6 (16.7)	8 (17.8)
Decreased	3 (8.3)	3 (6.7)
Discontinued	7 (19.4)	11 (24.4)
N/A^a^	19 (52.8)	17 (37.8)
Missing	0 (0)	5 (11.1)
Metformin, *n* (%)	n = 36	n = 45
Increased	0 (0)	2 (4.4)
No change	24 (66.7)	21 (46.7)
Decreased	9 (25.0)	8 (17.8)
Discontinued	1 (2.8)	5 (11.1)
N/A^a^	2 (5.6)	2 (4.4)
Missing	0 (0)	7 (15.6)

^a^Not taking at baseline

## Conclusion

This is the largest cohort of patients thus far described that had an EndoBarrier® implanted for 12 months. Significant reductions in weight, BMI and glycaemic control were observed during the device insertion period. The largest improvements for most parameters were noted within 3 months after device insertion, further but modest improvements in metabolic parameters continued between 3 and 12 months. Device insertion benefits were maintained at 6 months post-explant with small but non-significant metabolic parameter changes after explant.

Whilst the EndoBarrier® was in place, doses of both sulphonylureas and metformin were either reduced or, in a third of the subjects, the medications were discontinued. These reductions in pharmacotherapy coincided with reductions in HbA1c, fasting insulin and glucose. The largest and most significant reduction in weight was observed at 12 months suggesting continued negative energy balance whilst the device was implanted. This is in contrast to the usual plateau reached at 3–6 months with lifestyle and pharmacotherapy approaches.

Interestingly, the post-explant period did not show a prominent rebound effect or reversal of the metabolic benefits accrued during the period when the EndoBarrier® was in place.

### Safety

Published experience based on 271 EndoBarrier® implantations have shown the device to have a favourable safety profile [17]. In keeping with this, this study reported only one procedure-related event caused by the explant endoscope cap rather than the device itself. No other serious events were described. Outside the USA, serious adverse events included migration (4.9%), sleeve obstruction (3.4%) and liver abscess (0.126%). The U.S. EndoBarrier® clinical therapy (the ENDO Trial) was discontinued in July 2015 due to a 3.5% incidence of hepatic abscesses [16]. The aetiology of this has not been clearly established but may have resulted from localised seeding of infection from the foreign device to the liver or obstruction of the ampulla of Vater. This study reports no such events. Furthermore, the majority of the adverse effects reported were mild and the device well tolerated overall showing the EndoBarrier® to be both a safe and effective device.

### Limitations

This study reports the largest group of study participants with type 2 diabetes and obesity that have had the EndoBarrier® inserted. The results show a beneficial effect on weight loss and improvement in diabetes; however, the number of subjects is not sufficient to evaluate safety comprehensively and may be a limitation of the study. The results generated from data obtained from the completer population suggest the device is safe and effective over 12 months. Most of the literature to date outlines short-term results from smaller studies with limited follow-up. Our study reports mid-term outcomes, but future studies should include longer term outcomes and safety profiles for wider uptake of the device and establishment of the device efficacy. This study did not include a diet control group but it provides the effect size of benefits we can expect to allow a future randomised placebo-controlled trial to be powered appropriately.

Six out of the 14 patients who withdrew from the study required premature EndoBarrier® removal. Of these, only two patients presented with device-related complications (abdominal pain due to device migration and gastrointestinal bleeding); the others withdrew due to independent mainly vascular medical adverse events. Cardiovascular disease is the leading cause of mortality in patients with diabetes, with an increased risk of developing myocardial infarction, peripheral vascular disease, stroke and heart failure [18]. Tight glycaemic control has been shown to improve microvascular diabetes-related complications; however, similar improvements in primary cardiovascular events have not been demonstrated [19, 20]. This suggests this population remains at risk of cardiovascular events in the short term despite improvements in glycaemic control following EndoBarrier® insertion. Unfortunately, this increased risk usually requires prophylactic antiplatelet therapy, a contraindication to device insertion rendering the patient unsuitable for EndoBarrier® and limiting the wider uptake of the device.

One patient developed deep vein thrombosis requiring anticoagulation therapy, also a contraindication to device insertion and hence required premature explant. Patients with diabetes have been shown to be at increased risk of thromboembolic events [21] possibly as a result of impaired fibrinolysis and plasma hypercoagulability. Confounding risk factors also present in this population include higher BMI, dyslipidaemia and inflammation which will continue to increase the risk of thromboembolic events, at least in the short-term after device insertion. Additionally, although all patients in this study had straightforward implantation procedures, it is important to note that there are patients with a short duodenal bulb length in whom the device may not be inserted securely and be at risk of device migration, rotation and failure. Finally, medication was discontinued as a safety precaution as to prevent hypoglycaemia, but future studies may aim to continue drugs like metformin to obtain better glycaemic control.

For patients, health care providers and commissioners alike, a clearer understanding of the mechanisms involved in improving glycaemia after such interventions is essential and better data is required. The EndoBarrier® appears to work by allowing bile to have undiluted contact with the proximal small bowel mucosa, blocking food having contact with the proximal small bowel mucosa and by changes in gut flora and gut hormones. There are a number of potential metabolic consequences of the device insertion such as an increase in satiation potentially due to changes in gut hormones such as PYY, GLP-1 and oxyntomodulin. Other potential changes may include increases in diet-induced thermogenesis and change in taste and food preference. Further studies could investigate whether the EndoBarrier® confers a specific metabolic advantage in patients with type 2 diabetes by assessing whether and to what extent it replicates the BRAVE effects of bariatric surgery and its downstream sequelae, and how this might improve glucose-mediated insulin release and/or reducing liver-specific insulin resistance. The efficacy of the device for each obesity class could also be determined in future studies.

Increasingly, patients and physicians are looking towards minimally invasive techniques as an alternative to conventional surgery to treat chronic diseases. The EndoBarrier® is a device easily implanted and explanted without incisions, reducing complications, time, cost and recovery associated with conventional treatment modalities. Any measures to reduce these whilst maintaining or improving the patient treatment experience and post-procedure quality of life by the omission of surgery should be further explored. This study shows the EndoBarrier® to be a safe and effective device for the treatment of obesity associated with type 2 diabetes.
